# Commentary: “Hearing faces and seeing voices”: Amodal coding of person identity in the human brain

**DOI:** 10.3389/fnins.2017.00303

**Published:** 2017-05-30

**Authors:** Nadine Lavan

**Affiliations:** Department of Psychology, Royal Holloway, University of LondonLondon, United Kingdom

**Keywords:** person identity, amodal processing, MVPA, cross-classification, Identity representations

In a recent paper, Hasan et al. (2016) report the results of a neuroimaging study on amodal person identity processing. Across multiple testing sessions, five participants were presented with audio-only, video-only and audiovisual stimuli of four familiar people producing the syllable “had.” During the scanning session, participants performed a forced-choice person identification task in response to each stimulus. A univariate fMRI analysis confirmed that visual and auditory cortices where involved in stimulus processing. Using multivoxel pattern analyses (MVPA), the authors found that auditory identities could be successfully classified in temporal areas, while visual identities could be successfully classified in fusiform gyrus and inferior temporal gyrus. In addition, auditory and visual identities could both be classified in overlapping areas of right posterior superior temporal sulcus (pSTS). Successful *cross-classification* of stimuli was achieved in left inferior frontal gyrus, right supramarginal gyrus and in multiple sites in STS—here classifiers were trained on data from one modality and tested on data from the other, thus probing modality-independent coding. Hasan et al. (2016) conclude that their results are evidence for increasingly abstracted neural representations of person identity. This study addresses important theoretical questions about the multimodal processing of person identity and attempts to empirically map identity coding from unimodal toward abstracted amodal representations. However, several issues arise from the study design and interpretation.

## Generalisability and dependencies between modalities

Hasan et al. (2016) use a relatively small number of stimuli (12 in total; 4 identities × 1 stimulus per modality [audio-only, video-only, audiovisual]). Due to the design of the experiment, each condition was therefore only tested using a single stimulus. It is consequently unclear whether successful classifications within each modality are stimulus-specific effects or whether they would generalize across other (sets of) stimuli representing the same identities—a concern already noted by the authors. Further issues arise from deriving the audio-only and video-only stimuli from the same original audio-visual stimulus: cues to amplitude, vowel quality and even features of the facial expression of a person are encoded in both the auditory and visual channels (Summerfield, 1991; or see the impact of smiling on vocalizations; Ohala, 1980; Aubergé and Cathiard, 2003). The audiovisual, audio-only and video-only stimuli for each identity were thus themselves not independent of each other and, crucially, shared information unrelated to person identity. Due to these stimulus-specific dependencies, the authors' interpretation that successful cross-classification is “‘true’ identity classification, as opposed to *stimulus* classification” (p. 5) is problematic: The results of the cross-classification may still at least partially reflect stimulus effects. The current study thus shows no clear support for modality-dependent (due to a lack of evidence for generalization) or modality-independent (due to stimulus dependencies) identity classification as such.

## Identity-specific coding?

It further remains unclear whether Hasan et al. (2016) have shown evidence for the coding of familiar identities as opposed to generic crossmodal coding. Cross-classification could be attributed to general effects of (previously learnt) associations between any familiar auditory and visual stimuli independent of the content of the stimuli (e.g., a hammer hitting an anvil) and may thus not be specific to person identity processing (see Kaplan et al., 2015 for a review of cross-classification studies). Further, the cross-classification results cannot be conclusively attributed to *familiar* person recognition (only) but could also be at least partially attributed to generic paired association learning: the study only included a small number of stimuli and each stimulus was presented on average 58 times per participant within the experiment, with the content of audio-only and video-only stimuli overlapping with that of the audiovisual stimuli. In such a design, associations between the stimuli representing the modalities could be formed, even for unrelated pairs of stimuli (e.g., a car paired with the sound of a bell), potentially resulting similar classification outcomes: Tanabe et al. (2005), for example, show that humans can (a) learn pairs of previously unfamiliar auditory and visual stimuli through limited exposure and that (b) this learning modulates (co-)activation of auditory and visual cortices across modalities (as measured in an univariate analysis) and in (posterior) STS. In the current study, paired association learning and identity processing cannot be conclusively disentangled. Hasan et al.'s (2016) study may serve as a proof of concept showing that the stimuli of the different identities were perceived to be distinct from each other—whether these distinctions were based on familiar identity processing or unknown features cannot be determined from the data. Thus, without having an adequate control condition to rule out that the processes reported here reflect generic crossmodal processing of any type of object or effects of paired association learning, interpreting the results as being reflective of abstracted identity-specific processing without considering other potential influences is difficult.

## Future directions

While there are several methodological and conceptual issues in Hasan et al.'s (2016) study, the study nonetheless offers valuable new insights into how multivariate analyses of neuroimaging data can be harnessed to further our understanding of person identity processing. In contrast to the univariate approaches that were used in most previous studies looking at identity processing, multivariate analyses take into account information encoded in distributed spatial patterns of neural activity (Haynes and Rees, 2006). These methods thus provide intriguing possibilities to ask and address novel questions about neural representations of person identity in a nuanced way and can offer complimentary findings to what has already been established by studies using univariate analyses. These questions can offer insights into what the structure and content of representations of person identity at different processing stages is. They can furthermore offer insights into how different modalities and other sources of (context-dependent) information may interact, and into how variable information from multiple modalities is generalized into abstract representations. Within the constraints of these new methods, it is however essential to take care in choosing adequate stimulus materials and study designs that go beyond stimulus effects and truly tap into the abstracted identity representations.

## Author contributions

The author confirms being the sole contributor of this work and approved it for publication.

### Conflict of interest statement

The author declares that the research was conducted in the absence of any commercial or financial relationships that could be construed as a potential conflict of interest.
